# EEG-driven automatic generation of emotive music based on transformer

**DOI:** 10.3389/fnbot.2024.1437737

**Published:** 2024-08-19

**Authors:** Hui Jiang, Yu Chen, Di Wu, Jinlin Yan

**Affiliations:** ^1^School of Computer Science and Artificial Intelligence, Hefei Normal University, Hefei, China; ^2^School of Computer Science and Artificial Intelligence, Wuhan Textile University, Wuhan, China; ^3^Electricnic Information Engineering, Xi'an Technological University, Xi'an, China

**Keywords:** EEG, emotive music generation, transformer, latent features, information retrieval

## Abstract

Utilizing deep features from electroencephalography (EEG) data for emotional music composition provides a novel approach for creating personalized and emotionally rich music. Compared to textual data, converting continuous EEG and music data into discrete units presents significant challenges, particularly the lack of a clear and fixed vocabulary for standardizing EEG and audio data. The lack of this standard makes the mapping relationship between EEG signals and musical elements (such as rhythm, melody, and emotion) blurry and complex. Therefore, we propose a method of using clustering to create discrete representations and using the Transformer model to reverse mapping relationships. Specifically, the model uses clustering labels to segment signals and independently encodes EEG and emotional music data to construct a vocabulary, thereby achieving discrete representation. A time series dictionary was developed using clustering algorithms, which more effectively captures and utilizes the temporal and structural relationships between EEG and audio data. In response to the insensitivity to temporal information in heterogeneous data, we adopted a multi head attention mechanism and positional encoding technology to enable the model to focus on information in different subspaces, thereby enhancing the understanding of the complex internal structure of EEG and audio data. In addition, to address the mismatch between local and global information in emotion driven music generation, we introduce an audio masking prediction loss learning method. Our method generates music that *Hits@*20 On the indicator, a performance of 68.19% was achieved, which improved the score by 4.9% compared to other methods, indicating the effectiveness of this method.

## 1 Introduction

With the advancement of technology, especially the rapid development of artificial intelligence, significant changes have occurred in the field of music production. Artificial intelligence algorithms and other digital tools not only accelerate the process of music creation, but also open up new means of creation and expression for musicians. Traditional composition involves composers using music theory and personal creativity to create melodies, harmonies, and rhythms (Liu, 2023a). Electronic music production utilizes computer software such as electronic instruments and digital audio workstations (DAW) to create and edit music using virtual instruments, samples, and synthesizers. Algorithm music involves automatically generating music through specific algorithms or mathematical models, which can generate melodies and harmonies based on preset rules or randomness (Zeng and Zhou, 2021). Artificial intelligence music generation uses technologies such as machine learning and deep learning to extensively analyze music data, learn music styles and structures, and create new works (Bitaraes et al., 2022). The introduction of AI is not intended to replace traditional music creation methods or musicians, but to enhance their creative abilities and stimulate new ideas.

Previous studies have typically used techniques such as Long Short Term Memory (LSTM) and Recurrent Neural Networks (RNN) to achieve music generation. For example, Zhao et al. (2019) utilized biLSTM (bidirectional LSTM) to generate polyphonic music and designed a controllable parameter system based on Russell's two-dimensional emotional space (pleasure arousal). The system introduced the concept of LookBack to improve long-term structure, aiming to generate music that expresses four basic emotions. Dua et al. (2020) used Recurrent Neural Networks (RNNs), Gated Recursive Units (GRUs), and Long Short Term Memory Units (LSTMs) to improve the source separation and chord estimation modules, thereby enhancing the accuracy of score generation. Minu et al. (2022) regards music generation as a sequence to sequence modeling task, utilizing recursive neural networks to capture long-term time structures and patterns present in music data. Neural networks analyze a large amount of music data to learn and simulate the characteristics of specific music styles, thereby generating new music works. However, these algorithms still face challenges in understanding and generating complex music structures and their emotional expressions, especially in capturing subtle changes in music structures and deep emotions.

The application of emotion recognition and expression technology in music creation. This is mainly achieved through two optimization methods: first, creating a dataset with different emotional annotations, allowing the model to learn and imitate rich emotional expressions during the training process. Secondly, through interdisciplinary cooperation, integrating knowledge and technology from different fields, we can more effectively address the challenges of emotional expression in music. Hung et al. (2021) developed a shared multimodal database called EMOPIA, focusing on perceptual emotions in popular piano music, including audio and MIDI formats. Zheng et al. (2022) introduced an emotionBox method driven by music elements based on music psychology. The method extracts pitch histograms and note densities to represent pitch and rhythm, respectively, to control the emotional expression of music. Ma et al. (2022) proposed a new method for synthesizing music based on specific emotions, embedding emotional labels and music structure features as conditional inputs, and applying GRU networks to generate emotional music. However, accurately conveying human emotions to models remains a major challenge, especially in ensuring that the complexity and multidimensional nature of emotions are fully understood and expressed.

With the rapid development of Transformer technology, researchers have begun to effectively utilize data from large music libraries to analyze and learn the rhythm and melody of music through methods such as self supervised learning and transfer learning. Liu et al. (2022) developed a model of music motion synchronous generative adversarial network (M2S-GAN) to automatically learn music representations and generate command actions. This model can simulate the key functions of human conductors in understanding, interpreting, accurately, and elegantly performing music. Zhang and Tian (2022) developed a dual Seq2Seq framework based on reinforcement learning, which establishes a reward mechanism for emotional consistency and content fidelity to ensure that the generated melody is consistent with the emotional content of the input lyrics. Latif et al. (2023) proposed an Adversarial Dual Discriminator (ADDi) network, which uses a three person adversarial game to learn generalized representations and self supervised pre training using unlabeled data. Abudukelimu et al. (2024) developed a symbol music generation model called SymforNet based on self supervised learning, which utilizes attention mechanisms to achieve excellent ability to recognize different contextual elements. At present, these technologies mainly rely on methods that generate similar content based on specific text or music segments, and the generated music may not meet user expectations in terms of emotional expression.

An increasing number of studies are using auxiliary information to generate emotional music rather than relying on random generation. For example, techniques like Riffusion (Forsgren and Martiros, 2022) and Mubert (Mubert-Inc, 2022) use text information. Riffusion (Forsgren and Martiros, 2022) converts text-to-audio generation into spectrogram images and uses diffusion methods to maintain the stability of the music style. Mubert (Mubert-Inc, 2022) encodes text prompts and labels into latent vectors and selects the most matching labels to generate music. EEG signals are objective records of how people experience music, reflecting the impact of music on the brain. There is a general correlation between EEG signals triggered by music video stimuli and the content of the music video, and this information can effectively guide music generation. Therefore, utilizing EEG signal data to drive the generation of emotional music is of significant importance. Due to the lack of unified standards and classification methods, the mapping relationship between EEG signals and musical elements (such as rhythm, melody, and emotion) has become blurred and complex (Matsuda and Yamaguchi, 2022). EEG typically contains high levels of noise and is influenced by individual physiological and psychological states, while music data needs to address the complexity of polyphonic structures, volume changes, and different styles and genres (Bellisario et al., 2017). There are two main issues, one is the lack of a clear and fixed vocabulary between EEG and music data to standardize information. The other, the challenges posed by their respective data problems make it difficult to map between modalities.

To address these challenges, this study proposes a new approach that utilizes clustering to construct discrete representations and effectively utilizes complex and continuous EEG data to generate music for emotional expression through a Transformer model reverse mapping relationship. To address the mismatch between local and global information in emotion driven music generation, we introduce audio masking prediction loss learning technology to learn the global features of audio data. The model can more effectively understand and integrate the direct and overall emotional context, making music works more emotionally coherent and bridging the gap between short-term music elements and the overall emotional curve.

This study conducted extensive experiments on the EEG emotion dataset and demonstrated the following points:

1. Our model effectively integrates EEG and music data, and applies positional encoding to create robust discrete representations. Through the Transformer reverse mapping relationship, it significantly enhances the potential connection between EEG signals and audio data, and improves the emotional correlation of generated music.

2. The use of audio masking prediction loss forces the model to learn long sequence contextual information, enhancing its ability and accuracy in processing global information in music, ensuring that the generated music reflects a coherent and comprehensive emotional narrative, closely integrated with EEG driven emotional cues.

3. Our method performed well on multiple evaluation metrics and achieved the best results. This indicates that the generated music not only resonates well with the expected emotional state, but also maintains a high level of music quality.

## 2 Related work

### 2.1 The definition of beautiful music

Music is often regarded as beautiful because it has the ability to evoke emotions, aesthetic appreciation, or pleasure among listeners. Beautiful music typically possesses qualities such as harmony, melody, rhythm, creativity, and emotional expression. Through the harmonious combination of various musical notes, it generates pleasing harmonies and clear melodic lines, as well as a strong sense of rhythm, effectively conveying emotions and eliciting resonance among listeners (Bruns et al., 2023).

Emotional music generation can be divided into three main approaches: style transfer (Liu, 2023b), symbolic-level generation (Zhou et al., 2023b), and music snippet generation (Wang and Yang, 2019). Style transfer involves transforming existing music pieces into new ones with specific styles or emotional characteristics. This process involves analyzing the style features of the original piece and incorporating these features into the new piece, such as transforming a classical music piece into a jazz style. Symbolic-level generation uses symbolic data (such as MIDI files) instead of directly processing audio waveforms to create music. By applying models like LSTM to capture the temporal characteristics of music, melodies and chords can be generated. Additionally, by adding emotional labels to the input symbolic music data, the model can learn and generate music with corresponding emotions. Music snippet generation involves creating short, clearly emotional or stylistic music snippets, which may form part of a complete composition or stand alone as independent musical phrases. This process often employs deep learning algorithms, including Recurrent Neural Networks (RNNs), Variational Autoencoders (VAEs), and Generative Adversarial Networks (GANs). These algorithms adjust rhythm and pitch by directly processing audio samples, thereby generating music snippets with specific emotions. Particularly, GANs and VAEs can learn complex data distributions and create both natural and expressive new music snippets. The understanding of the beauty of music is highly subjective and varies with cultural and individual differences. Nevertheless, it is generally believed that music capable of eliciting deep emotional resonance has universal aesthetic value.

### 2.2 Characteristic representation of brain electricity

EEG is a method for recording electrical activity in the brain, widely used in studies of brain function and conditions. By analyzing EEG data, researchers can obtain characteristics of brain electrical activity and interpret rich information therein (Sánchez-Reyes et al., 2024). This non-invasive technique can be employed to study brain function, cognitive processes, sleep states, and neurological disorders, among others (Özdenizci et al., 2020). By analyzing the spectrum, coherence, and temporal parameters of EEG signals, interactions between brain regions can be revealed, and the functioning of the brain under specific tasks or states can be explored. Additionally, EEG data analysis aids in a deeper understanding of brain activity patterns underlying cognitive processes. Focusing on changes in specific frequency bands and features of event-related potentials helps in understanding the brain's response to different cognitive tasks, such as attention, memory, and language processing. Moreover, EEG is also used to monitor changes in brain activity during the treatment of neurological disorders or disease progression. By combining different EEG features with advanced data analysis techniques and machine learning algorithms, researchers can delve deeper into EEG signals, providing comprehensive insights into understanding brain activity mechanisms, brain functional disorders, and research on brain-computer interfaces, thus driving advancements in neuroscience and clinical diagnosis and treatment of neurological diseases.

### 2.3 Clustering to build dictionary table

Clustering methods, dictionary construction, and high-dimensional data embedding are crucial tools in the fields of data analysis and machine learning, playing pivotal roles in handling vast datasets. In natural language processing (NLP) tasks, word embedding techniques offer rich text representations by capturing similarities and contextual relationships among words (Krishnan and Jawahar, 2020). Simultaneously, dictionary construction ensures consistency in text processing, which is vital for applications such as text classification and sentiment analysis. For instance, Gozuacik et al. (2023) utilized word embedding techniques to generate word vectors and employed LSTM networks to simulate the temporal evolution of word associations, establishing a model for predicting word embedding matrices. High-dimensional data embedding techniques encode raw data into feature vectors, effectively preserving key information and structure. After undergoing intermediate layer network mapping and decoding processes, they can accurately extract the desired information features. Clustering, as an unsupervised learning technique, groups data into clusters or clusters based on the similarity between data points (Cai et al., 2023). The K-means clustering method groups data by minimizing the sum of intra-cluster distances, while hierarchical clustering methods build hierarchical structures by progressively merging or splitting data points. Additionally, the Density-Based Spatial Clustering of Applications with Noise (DBSCAN) method identifies clusters of various shapes through density estimation and can effectively handle outliers. To achieve synchronized representations of music and EEG signals, we adopted a feature clustering approach to construct a discrete feature dictionary and established a model to map the relationships between these features.

### 2.4 Pre-trained model

More and more researchers are adopting pre-trained models to learn features from different modalities and apply them to downstream tasks, such as generating content for the target modality. In terms of task design, the Vision Transformer (ViT) directly applies the transformer architecture to sequences of image patches. Al-Quraishi et al. (2022) utilized Continuous Wavelet Transform (CWT) to generate time-frequency representations for each EEG signal and used the extracted images as inputs to deep learning models for classification. HuBERT is a model specifically designed for self-supervised learning of speech representations, which builds upon the architecture of BERT and trains by iteratively refining hidden representations. Seq2Seq, on the other hand, is a model architecture used for transforming one sequence into another, such as translating text from one language to another. Zhang et al. (2022) introduced self-supervised contrastive pre-training for time series data, including EEG signals, aiming to improve the performance of various downstream tasks. Panchavati et al. (2023) developed a transformer-based model pre-trained on annotated EEG data for seizure detection. Meanwhile, Zhou et al. (2023a) utilized pre-trained speech features to enhance EEG signal recognition, demonstrating the versatility of pre-trained models in various signal processing tasks. Using a transformer-based architecture to generate music based on EEG inputs is an innovative approach. Transformers are particularly effective in handling sequence data with long-range dependencies, making them suitable for modeling complex EEG signal patterns related to music.

## 3 Methods

**Figure 1:**
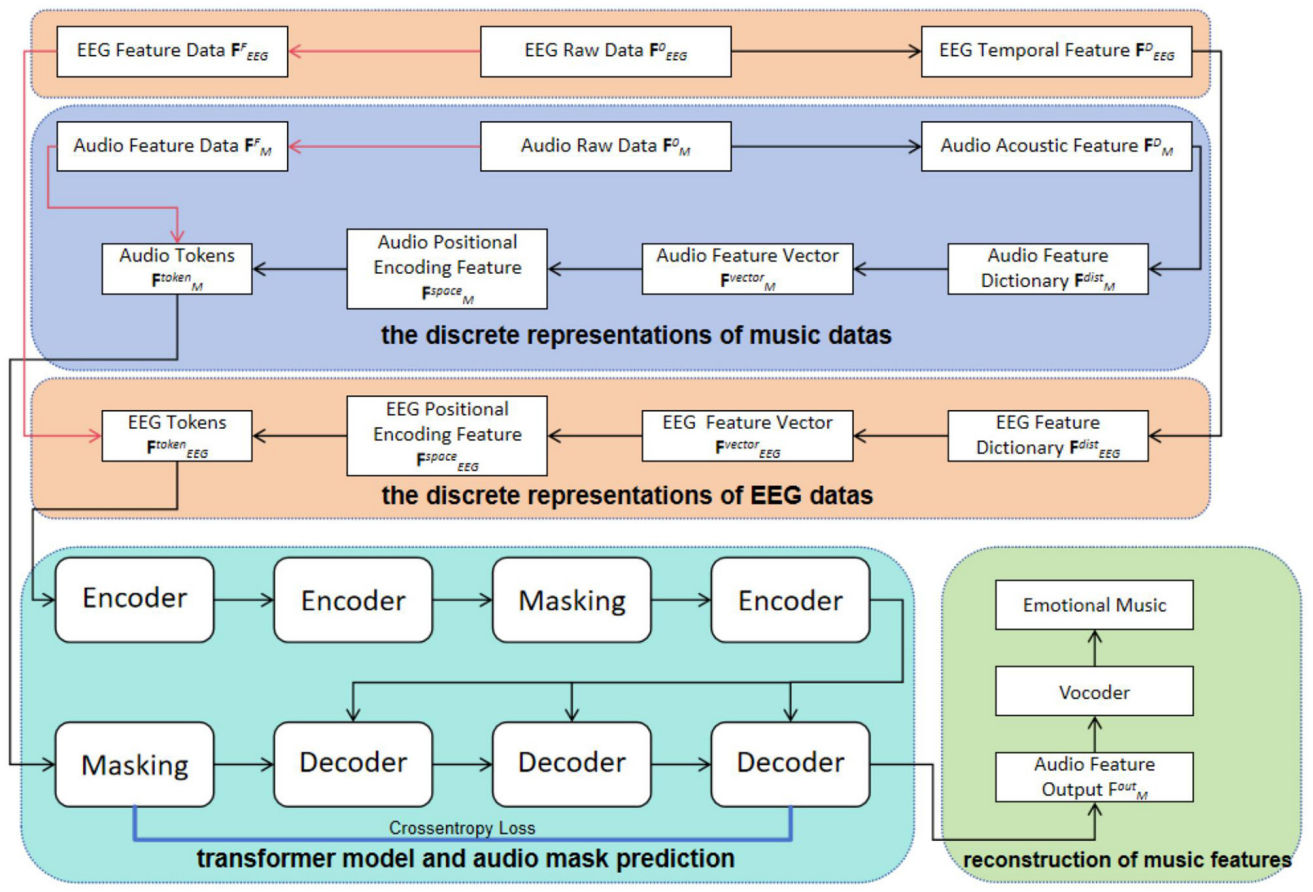
The overall framework consists of four steps: discrete representations of music datas, discrete representations of EEG datas, transformer model and audio mask prediction, and reconstruction of music features. Initially, convolutional feature extraction is applied to the filtered and aligned EEG raw data, denoted as ${\text{F}}_{EEG}^{0}$, and the raw audio data, denoted as ${\text{F}}_{M}^{0}$ (indicated by the red arrows). Subsequently, a series of feature transformation operations are performed on ${\text{F}}_{EEG}^{0}$ and ${\text{F}}_{M}^{0}$ to construct spatial encoding features, ${\text{F}}_{EEG}^{space}$ and ${\text{F}}_{M}^{space}$, and generate EEG tokens, ${\text{F}}_{EEG}^{token}$, and audio tokens, ${\text{F}}_{M}^{token}$. These tokens are then inputted into a Transformer architecture for inter-token feature mapping, ultimately resulting in the audio feature, ${\text{F}}_{M}^{out}$, which is utilized for reconstructing emotional music.

EEG data and music audio data represent different dimensions of information, and thus we processed each type separately. In handling the EEG data, we first performed downsampling and filtering, followed by data alignment and slicing, eventually obtaining multi-channel aligned feature vectors ${\text{F}}_{EEG}^{0}\in {\mathbb{R}}^{N\times M}$, known as the raw EEG data. Here, *N* represents the number of channels, while *M* represents the sample length. As for the music audio data, we extracted the music feature ${\text{F}}_{M}^{0}\in {\mathbb{R}}^{1\times M}$ through appropriate audio processing techniques, referred to as emotional music data, where *M* also indicates the sample length. This processing approach ensures the consistency and comparability of the two different types of data. As shown in Figure 1, we present the detailed steps of the entire processing workflow. In this study, both EEG data and emotional music data are simultaneously input into our training model. The purpose of this is to develop a new model that utilizes both types of data, thereby achieving the task of generating emotional music expressed through brainwave signals.

When processing the ${\text{F}}_{EEG}^{0}$ data, we first extracted the differential entropy features and applied the DBSCAN density clustering algorithm for adaptive learning to obtain a cluster set, which further refined the EEG's temporal feature ${\text{F}}_{EEG}^{D}$. Based on these temporal features, we constructed an EEG feature dictionary ${\text{F}}_{EEG}^{dist}$, from which the EEG feature vector ${\text{F}}_{EEG}^{vector}$ were derived. Additionally, we acquired ${\text{F}}_{EEG}^{space}$ through a positional encoding function, providing richer spatiotemporal information for model training. In handling the emotional music data, we used a method similar to the EEG signal processing. First, we utilized the DBSCAN density clustering algorithm for adaptive learning to obtain a cluster set of emotional music, and then sequentially extracted the features ${\text{F}}_{M}^{D}$, ${\text{F}}_{M}^{dist}$, ${\text{F}}_{M}^{vector}$, and ${\text{F}}_{M}^{space}$. For the EEG signals, ${\text{F}}_{EEG}^{F}$ was obtained by applying a Convolutional Neural Network (CNN) to the raw EEG data ${\text{F}}_{EEG}^{0}$ for feature mapping. Subsequently, we combined ${\text{F}}_{EEG}^{F}$ and ${\text{F}}_{EEG}^{space}$ to construct EEG tokens ${\text{F}}_{EEG}^{token}$. Similarly, the audio feature data ${\text{F}}_{M}^{F}$ was obtained by mapping the audio raw data ${\text{F}}_{M}^{0}$ through a CNN network. Afterwards, ${\text{F}}_{M}^{F}$ was combined with ${\text{F}}_{M}^{space}$ to construct audio tokens ${\text{F}}_{M}^{token}$.

The EEG tokens ${\text{F}}_{EEG}^{token}$ and audio tokens ${\text{F}}_{M}^{token}$ are input into a model based on the Transformer architecture for deep feature interaction, thereby realizing the potential feature expression and mapping relationship between them. The self-attention mechanism of the Transformer model can effectively capture the complex dependencies between tokens, thereby extracting high-quality features that reflect the interaction between brainwave signals and musical emotions.

### 3.1 Clustering to build discretized representations

In this section, we elaborate on how clustering methods are utilized to construct discretized representations for generating tokens from EEG and audio data. Initially, we extract differential entropy features from the original continuous feature data and apply the DBSCAN clustering algorithm to effectively group these continuous features into meaningful categories. Subsequently, we assign a unique identifier to each category and construct a feature dictionary. Through this process, we transform the original continuous features into a series of discrete feature vectors, thereby achieving a mapping from continuous features to discrete feature sequences. Following these steps, we effectively transform the original continuous features into discretized representations and, based on these representations and the original features, construct tokens. These tokens can then be input into the Transformer model for deep learning and interaction between features.

#### 3.1.1 DBSCAN clustering method

Density-Based Spatial Clustering of Applications with Noise (DBSCAN) is a density-based spatial clustering algorithm capable of identifying clusters of arbitrary shapes and effectively handling noise data. The core idea of the algorithm is to cluster based on the density of the area where objects are located. In the DBSCAN algorithm, there are two key parameters: radius ϵ and minimum number of points MinPts. These parameters determine whether an area is dense enough to form a cluster. The operation of DBSCAN can be summarized in the following steps: First, the algorithm starts with any unprocessed point as the starting point and explores its ϵ-neighborhood; if the number of points in this neighborhood reaches MinPts, the point is marked as a core point, otherwise, it is marked as a boundary point or noise point. Then, starting from a core point, the algorithm adds all points in the neighborhood to the cluster and recursively expands the neighborhood of all newly added core points.

We define the sequence ${\text{F}}_{D}^{0}$, represented as ${\text{F}}_{D}^{0}=\left\{f_{1},f_{2},\ldots ,f_{M}\right\}$, where each element *f*_*i*_ represents a feature in the data. Using these features, the DBSCAN algorithm groups data points into clusters such that the distance between any two points within a cluster is less than the set radius ϵ, and each cluster contains at least MinPts points. The ϵ-neighborhood of a point is defined as the collection of all points within a circular area centered at the point with radius ϵ. For any point *f*_*i*_ in the sequence ${\text{F}}_{D}^{0}$, its ϵ-neighborhood can be represented by the following mathematical Equation 1 formula:


(1) $$\begin{array}{l} F_{DBSCAN}\left(f_{i}\right)=\left\{q\in {\text{F}}_{D}^{0}|\text{dist}\left(f_{i},q\right)\leq \epsilon \right\} \end{array}$$

Here, dist(*f*_*i*_, *q*) denotes the distance between the point *f*_*i*_ and point *q*. If the ϵ-neighborhood of the point *f*_*i*_ contains at least MinPts points (including *f*_*i*_ itself), then *f*_*i*_ is labeled as a core point. This condition can be represented by the following Equation 2 formula:


(2) $$\begin{array}{l} |F_{DBSCAN}\left(f_{i}\right)|\geq \text{MinPts} \end{array}$$

where, $\left|F_{DBSCAN}\left(f_{i}\right)\right|$ represents the number of points in the set $F_{DBSCAN}\left(f_{i}\right)$.

If a point is not a core point, and the number of points in its ϵ-neighborhood is less than MinPts, but it includes at least one core point within its *ϵ*-neighborhood, then that point is marked as a boundary point. Points that are neither core points nor boundary points are considered noise points. During the clustering process, if a point *f*_*i*_ is a core point, then any point *q* within its *ϵ*-neighborhood is considered to be directly density-reachable from *f*_*i*_. This relationship can be defined by the following Equation 3 condition:


(3) $$\begin{array}{l} q\in F_{DBSCAN}\left(f_{i}\right)\;\text{and }|F_{DBSCAN}\left(f_{i}\right)|\geq \text{MinPts} \end{array}$$

where the point *q* is part of the same cluster as *f*_*i*_, driven by the density connection that *f*_*i*_ establishes through its sufficient *ϵ*-neighborhood size. In this way, DBSCAN can effectively identify and distinguish clusters of various shapes and sizes in time series and handle noise points in the data.

#### 3.1.2 EEG signals build EEG tokens

In the process of constructing EEG tokens, we first perform feature extraction on the processed dataset ${\text{F}}_{EEG}^{0}$. We extract differential entropy features, which are defined by the function $F_{De}$. Then, using the density clustering algorithm DBSCAN, we perform adaptive learning on these differential entropy features to obtain the clustered EEG temporal features ${\text{F}}_{EEG}^{D}$. This process can be precisely described by the following Equation 4 formula:


(4) $$\begin{array}{l} {\text{F}}_{EEG}^{D}=F_{DBSCAN}^{EEG}\left(F_{De}\left({\text{F}}_{EEG}^{0}\right)\right) \end{array}$$

In the process of constructing the EEG feature dictionary ${\text{F}}_{EEG}^{dist}$, we first use the feature clusters obtained through the DBSCAN algorithm as the elements of the dictionary. Each cluster's centroid and its representative features together form an entry in the dictionary. This EEG feature dictionary ${\text{F}}_{EEG}^{dist}$ is used to represent and encode the features of EEG data. The EEG feature vector ${\text{F}}_{EEG}^{vector}$ is generated based on the similarity between the features of a new signal and the most matching entry in the dictionary. This process can be represented by the following Equation 5 formula:


(5) $$\begin{array}{l} {\text{F}}_{EEG}^{vector}=F_{dict}^{EEG}\left({\text{F}}_{EEG}^{D}\right) \end{array}$$

Here, $F_{dict}^{EEG}$ is a function that maps the clustered features ${\text{F}}_{EEG}^{D}$, obtained through clustering, to the EEG feature dictionary ${\text{F}}_{EEG}^{dist}$. This mapping facilitates the generation of the EEG feature vector ${\text{F}}_{EEG}^{vector}$ for EEG signals. Constructing the feature dictionary ${\text{F}}_{EEG}^{dist}=c_{1}:\left(centroid_{1},features_{1}\right),c_{2}:\left(centroid_{2},features_{2}\right),\ldots ,$ *c*_*k*_:(*centroid*_*k*_, *features*_*k*_), *c*_*i*_ represents the *i*th cluster, *centroid*_*i*_ is the centroid of the *i*th cluster, and *features*_*i*_ are the representative features of the *i*th cluster.

To fully leverage the spatiotemporal information in EEG signals, this study inputs the ${\text{F}}_{EEG}^{vector}$ signals into a positional encoding function to generate richer positional encoding feature ${\text{F}}_{EEG}^{space}$. This process can be described by the following Equation 6 formula:


(6) $$\begin{array}{l} {\text{F}}_{EEG}^{space}=F_{space}^{EEG}\left({\text{F}}_{EEG}^{vector}\right) \end{array}$$

where, $F_{space}^{EEG}$ is a function specifically designed to add spatial and temporal context to the feature vectors derived from the EEG signals. The Sinusoidal Position Encoding method uses sine and cosine functions to generate encodings for each position in the sequence. The purpose of this method is to provide the model with information about the positions in the sequence, helping the model to better understand the order characteristics of the sequence data. The position encoding function $F_{PE}$ is specifically defined as follows Equation 7:


(7) $$\begin{array}{l} F_{PE}\left(pos,k\right)=\left\{ \begin{array}{l} \sin\left(\frac{pos}{10,000^{2i/d_{model}}}\right),k=2i\\ \cos\left(\frac{pos}{10,000^{2i/d_{model}}}\right),k=2i+1 \end{array}\right. \end{array}$$

In this, *pos* represents the position index in the sequence, *k* is the dimension index of the positional vector, 2*i* and 2*i*+1 to distinguish the parity of *k*, and *d*_*m*_*odel* is the total dimension of the model. This encoding method assigns a sinusoid to each dimension, with wavelengths varying geometrically from 2π to 10, 000 × 2π.

This method is used to generate a unique encoding for each position in the EEG data. These encodings not only contain positional information but also reflect the similarities between similar positions. We construct the EEG positional encoding feature ${\text{F}}_{EEG}^{space}$ and the EEG feature data ${\text{F}}_{EEG}^{F}$ into EEG tokens ${\text{F}}_{EEG}^{token}$, where each token includes a comprehensive representation of both EEG features and positional information.

#### 3.1.3 Emotional music build audio tokens

For emotional music data, although audio data is typically single-channel, differing in dimension from multi-channel EEG signal data, a similar process can be used to construct audio tokens. This method transforms the time series data of music into tokens rich in information, making them suitable for deep learning models.

The audio raw data ${\text{F}}_{M}^{0}$ undergoes feature extraction, where features such as differential entropy could be extracted. These features are then adaptively learned using the DBSCAN algorithm to form clusters of audio acoustic feature ${\text{F}}_{M}^{D}$. This process can be represented as Equation 8:


(8) $$\begin{array}{l} {\text{F}}_{M}^{D}=F_{DBSCAN}^{M}\left(F_{De}\left({\text{F}}_{M}^{0}\right)\right) \end{array}$$

This step transforms continuous audio feature into discrete representations, similar to the method used for processing EEG signals.

Encode the clustering results from ${\text{F}}_{M}^{D}$ using the audio feature dictionary ${\text{F}}_{M}^{dist}$ to form the audio feature vector ${\text{F}}_{M}^{vector}$. Transform the clustering results into specific feature vectors through a mapping function Equation 9:


(9) $$\begin{array}{l} {\text{F}}_{M}^{vector}=F_{dict}^{M}\left({\text{F}}_{M}^{D}\right) \end{array}$$

Similar to EEG signals, perform positional encoding on the audio feature vector ${\text{F}}_{M}^{vector}$ using fixed formulas of sine and cosine functions to generate encodings for each position, which are the audio positional encoding feature ${\text{F}}_{M}^{space}$. This helps the model understand the temporal information of the audio data. This step can be represented as Equation 10:


(10) $$\begin{array}{l} {\text{F}}_{M}^{space}=F_{space}^{M}\left({\text{F}}_{M}^{vector}\right) \end{array}$$

The construction of audio tokens ${\text{F}}_{M}^{token}$ involves combining the position-encoded audio positional encoding feature ${\text{F}}_{M}^{space}$ with audio feature data ${\text{F}}_{M}^{F}$. These tokens include both the feature information of the audio and its temporal encoding, making them suitable for input into models for further analysis.

### 3.2 Transformer model

EEG signals and audio features first undergo preprocessing, including feature extraction, application of positional encoding, and feature dictionary conversion, to form the respective tokens ${\text{F}}_{EEG}^{token}$ and ${\text{F}}_{M}^{token}$. These tokens encode crucial information about the raw data and their position in the sequence, providing rich contextual information for the Transformer model.

The encoder layers receive the EEG tokens as input. Each encoder layer processes the input data through a self-attention mechanism and a feed-forward neural network, allowing each input to consider the influence of other inputs within the model, thus enhancing the model's understanding of the overall input data. Specifically, the processing of EEG signals also includes a feature mask label, which is based on the attention output from the last layer of the encoder. This label is used to enhance the expression of hidden features, enabling the encoder to acquire and generate richer semantic information, denoted as ${\text{F}}_{EEG}^{T}$.

Before the decoder starts processing, an audio mask is applied to the audio data. This audio mask prediction strategy requires the model to predict the masked parts. Through this approach, the model learns to use context to fill in missing information, improving its ability to process and accurately handle global information in music. The decoder layer utilizes self-attention mechanisms, encoder-decoder attention mechanisms, and a feed-forward network. The encoder-decoder attention mechanism allows the decoder layers to access the output of the encoder, helping the decoder better understand and predict the next steps of the output, thus achieving the audio feature output ${\text{F}}_{M}^{out}$.

We have adopted a Transformer model that transforms EEG signals and audio features into emotional music. Specifically, the model takes EEG signals and audio tokens as inputs and outputs a series of audio features used for music composition. After training, the Transformer model can generate audio features representing different musical elements, such as melody, rhythm, and harmony, based on EEG signals. Through a synthesizer, these audio features are effectively transformed into audio waveforms. Meanwhile, the synthesizer parameters are adjusted based on the emotional features in the EEG signals to produce music with specific emotional expressions.

### 3.3 Audio mask prediction

Audio mask prediction, as a method of data augmentation, primarily aims to extract and enhance valuable audio features from complex audio signals. By randomly masking sequences in the audio feature data ${\text{F}}_{M}^{F}$, and then defining these masked parts in the audio tokens ${\text{F}}_{M}^{token}$, as shown in Equation 11, it can effectively promote the model's learning and recognition of important features in audio data.


(11) $$\begin{array}{l} {\text{F}}_{M}^{mask}=F_{mask}^{M}\left({\text{F}}_{M}^{token}\right) \end{array}$$

After the masked datas ${\text{F}}_{M}^{mask}$ and ${\text{F}}_{EEG}^{T}$ are input into the decoder, the model outputs data ${\text{F}}_{M}^{de}$. This step is as shown in Equation 12.


(12) $$\begin{array}{l} {\text{F}}_{M}^{de}=F_{decoder}^{M}\left({\text{F}}_{M}^{mask},{\text{F}}_{EEG}^{T}\right) \end{array}$$

This process involves parsing and reconstructing the masked data to restore the parts that were masked in the original audio signal. The main task of mask prediction is to infer the missing audio information based on the known unmasked features and a comprehensive understanding of the audio data.

We adopt a composite loss function $F_{L}$ to balance different learning objectives during the training of the Transformer model, represented as Equation 13:


(13) $$\begin{array}{l} F_{L}=\left(1-\alpha \right)F_{L_{m}}+\alpha F_{L_{u}} \end{array}$$

where, $F_{L_{m}}$ is the loss for the masked portion, used to evaluate the model's performance in predicting the masked parts. $F_{L_{u}}$ is the loss for the unmasked portion, used to assess the model's performance in processing unmasked features. α is a tuning parameter used to balance the impact of these two types of losses. The formula for $F_{L_{m}}$ is expressed as Equation 14:


(14) $$\begin{array}{l} F_{L_{m}}=\underset{t\in T}{\sum }\log p\left(f_{t}|{\text{F}}_{M}^{mask},t\right) \end{array}$$

*T* represents the set of indices for the data after masking, the index *t* is used to extract the corresponding value from the feature ${\text{F}}_{M}^{mask}$, *f*_*t*_ is the *t*th position of the output ${\text{F}}_{M}^{de}$ a after the decoder, the symbol *p* represents the model's predicted probability distribution for that position.

This masking technique forces the model to rely on the context information it can observe to predict the missing parts of the data, significantly enhancing the model's learning and grasp of global audio features. This not only improves the model's ability to understand the structure of the data but also ensures the coherence and naturalness of the generated music in terms of listening experience. Through this method, we are able to develop an efficient model capable of capturing complex audio patterns and producing high-quality music.

## 4 Experiments

### 4.1 Datasets

The DEAP dataset (Koelstra et al., 2011) is important datasets used for studying the relationship between EEG signals and emotional responses. It consists of EEG signal recordings from 32 subjects. Subjects were recorded while watching 40 music videos, each lasting 1 min. The dataset was recorded using 32 EEG channels with a sampling frequency of 512 Hz, later downsampled to 128 Hz, and preprocessed with a bandpass filter ranging from 4 to 45 Hz. Each trial includes 3 s of baseline data and 60 s of experimental data. Participants were required to use a self-assessment manikin to evaluate the arousal, valence, liking, and dominance of each video.

The DEAP dataset focuses on the influence of music videos on emotional states and records EEG activity under visual and auditory stimuli. Table 1 represents the specific locations and channel names of the 32 channels in the DEAP dataset. It is particularly suitable for studying how music and visual content alter emotional states. Utilizing this dataset can assist researchers in understanding which music features are associated with specific emotional responses, thereby aiding in model training to generate music that evokes particular emotions.

**Table 1:** DEAP dataset 32 channel location and name.

**Channel no**.	**Ch. name Twente**	**Ch. name Geneva**	**Geneva > Twente**	**Twente > Geneva**
1	Fp1	Fp1	1	1
2	AF3	AF3	2	2
3	F7	F3	4	4
4	F3	F7	3	3
5	FC1	FC3	6	6
6	FC5	FC1	5	5
7	T7	C3	8	8
8	C3	T7	7	7
9	CP1	CP5	10	10
10	CP5	CP1	9	9
11	P7	P3	12	12
12	P3	P7	11	11
13	Pz	PO3	16	14
14	PO3	O1	13	15
15	O1	Oz	14	16
16	Oz	Pz	15	13
17	O2	Fp2	32	30
18	PO4	AF4	31	29
19	P4	Fz	29	31
20	P8	F4	30	27
21	CP6	F8	27	28
22	CP2	FC6	28	25
23	C4	FC2	25	26
24	T8	Cz	26	32
25	FC6	C4	22	23
26	FC2	T8	23	24
27	F4	CP6	20	21
28	F8	CP2	21	22
29	AF4	P4	18	19
30	Fp2	P8	17	20
31	Fz	PO4	19	18
32	Cz	O2	24	17

### 4.2 Experimental settings

#### 4.2.1 Data preprocessing

For EEG signals, segmenting the signal into 4-s intervals facilitates easier data processing, feature extraction, and analysis. This segmentation method can better capture changes and characteristics in the signal, making subsequent analysis more accurate and effective. Additionally, separating the music from the stimulus videos, isolating the reverberation components caused by environmental reflections, and separating vocals from background rhythmic music are crucial steps. This ensures that the music features are clearer, which is beneficial for subsequent tasks of generating emotional music rhythms and melodies. Such preprocessing steps allow for a better understanding of the emotional properties of music and provide a stronger foundation for generating music that elicits specific emotions. Lastly, dividing the subjects into control and experimental groups in an 8:2 ratio ensures that there are sufficient control samples during the experiment, facilitating better comparison and validation of results. This approach enhances the credibility and reliability of the research.

#### 4.2.2 Experiment details

We use *Hits@k* as a metric to evaluate our model. *Hits@k* is a metric used to assess the quality of search engine results, where *k* represents the top *k* results returned. *Hits@k* mainly focuses on recall and precision. Recall measures the coverage of relevant information in the retrieval results, i.e., whether the retrieval system can find all relevant information. A higher recall means that the system can find most of the relevant information, while a lower recall indicates that the system may miss some relevant information. Precision measures the accuracy of the retrieval results, i.e., how many of the results returned by the retrieval system are relevant. High precision means that most of the results returned by the system are relevant, while low precision indicates that the system may return many irrelevant results.

In the field of emotional music generation, *Hits@k* is a metric used to evaluate the generated chord progressions. It is typically used to calculate the proportion of reference chords present within the top *k* candidate chords, with common values of *k* being 1, 3, 5, 10, and 20. *Hits@k*, as illustrated in Equation 15, is a widely recognized evaluation metric for recommendation models and is pivotal in evaluating the effectiveness of emotional music generation models.


(15) $$\begin{array}{l} Hits@k=\frac{1}{n}\overset{n}{\underset{i=1}{\sum }}H\left({\text{rank}}_{i}\leq k\right), \end{array}$$

Here, H returns 1 if the correct answer ranks within the top *k* results, otherwise, it returns 0. It effectively measures the similarity and consistency between the generated chord progressions and the reference chords, thereby helping to evaluate the accuracy and quality of the model in generating emotional music.

#### 4.2.3 Baseline model

We conducted comparative experiments with the latest existing similar studies, specifically including RNN (Grekow, 2021), LSTM (Hizlisoy et al., 2021), (Tiraboschi et al., 2021), (Miyamoto et al., 2022), and Inoue (Inoue, 2024). RNN (Grekow, 2021) and LSTM (Hizlisoy et al., 2021) are capable of capturing long-term dependencies in time series data, hence existing methods for sentiment-based music generation typically employ these architectures. In our comparison, we replaced the transformer component with RNN and LSTM while keeping other aspects of our methodology unchanged, and still employed full-channel comparison. Tiraboschi et al. (2021) present an EEG-driven real-time music generation approach, utilizing OpenViBE for BCI and evaluating supervised learning methods for affect estimation. Additionally, it proposes modifications to Magenta's MusicVAE and introduces a Probabilistic Graphical Model to map affective states to music features. Here, we utilized the same channels as the original paper: FC5, FC6, CP5, CP6, PO3, and PO4. Miyamoto et al. (2022) introduce an individualized emotion induction system utilizing EEG-predicted emotions to generate tailored music. It comprises a music generator, real-time EEG emotion prediction, and music generator control, demonstrating effectiveness in inducing emotions and highlighting the potential of personalized stimuli for emotional induction. Here, we utilized the same 29 channels as in the original paper, which are: Fp1, Fp2, AF3, AF4, F7, F8, F3, Fz, F4, FC5, FC6, T7, T8, C3, Cz, C4, CP5, CP6, P7, P8, P3, Pz, P4, PO7, PO8, PO3, PO4, O1, O2. Inoue (2024) explore real-time EEG-driven music generation by employing EEG data collected from participants exposed to pre-measured music pieces. Utilizing a Support Vector Machine (SVM) and Emotiv Insight headset, EEG data was analyzed during the listening process of Beethoven's No. 6 and Barber's Adagio for Strings, focusing on arousal-valence impressions. Here, we used the same channels as in the original paper: AF3, AF4, T7, T8, and Pz.

### 4.3 Performance comparison

Based on the experimental results obtained from Table 2 of emotional music generation, we conducted predictive analysis using *k* = 1, 3, 5, 10 and 20. In these scenarios, compared to the optimal experimental results by Miyamoto et al., our method demonstrated improvements of 1.82, 1.91, 4.4, 6.56, and 4.90%, respectively. The experiments indicate that our method excels in all aspects, particularly demonstrating outstanding performance at *k* = 10. Based on the experimental results of *Hits@k*, our research indicates that our method exhibits significant advantages over other models at different values of *k*. This suggests that our method demonstrates higher accuracy and reliability in generating chord progressions compared to other models. Our method performs best at *k* = 10, with an improvement of 6.56%. This is because, at higher *k* values, the model has more options and can more accurately match the emotional chords progression in music. According to music generation theory, when the model has a larger sample space, it can better capture complex musical patterns, thereby generating chord progressions that better align with human emotional expectations. Despite the overall excellent performance, the improvement slightly decreases at *k* = 20 (4.90%). This could be due to the sample space being too large, leading to interference from some irrelevant or noisy data, which in turn reduces the model's accuracy. This suggests that when choosing the *k* value, we need to balance the selection space and noise interference to ensure the quality of the generated results. This finding holds important implications for music generation tasks, hinting at significant progress in our method's ability to emulate human composers' chord progressions.

**Table 2:** Experimental results of *Hits@k* for different models.

**Model**	***Hits@*1(*%*)**	***Hits@*3(*%*)**	***Hits@*5(*%*)**	***Hits@*10(*%*)**	***Hits@*20(*%*)**
RNN	18.56	28.37	36.25	45.24	54.39
LSTM	20.13	28.34	35.25	46.18	49.12
Tiraboschi et al.	18.42	26.24	37.24	45.72	56.25
Miyamoto et al.	22.45	35.52	40.89	50.68	63.29
Inoue	12.52	23.14	36.46	43.53	53.52
Ours	**24.27**	**37.43**	**45.29**	**57.24**	**68.19**

Bold text indicates the best values.

### 4.4 Quantitative analysis

**Figure 2:**
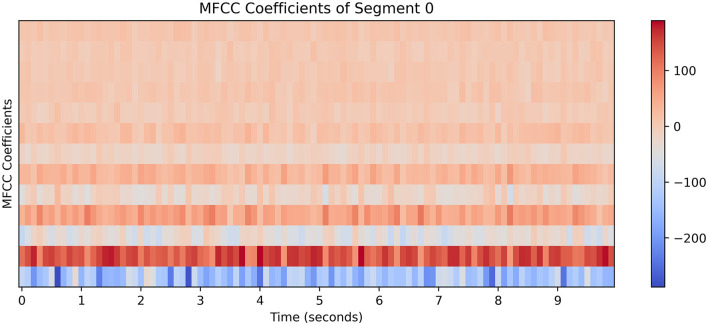
MFCC changes over time in segment 0, it shows the variation of MFCC over time.

**Figure 3:**
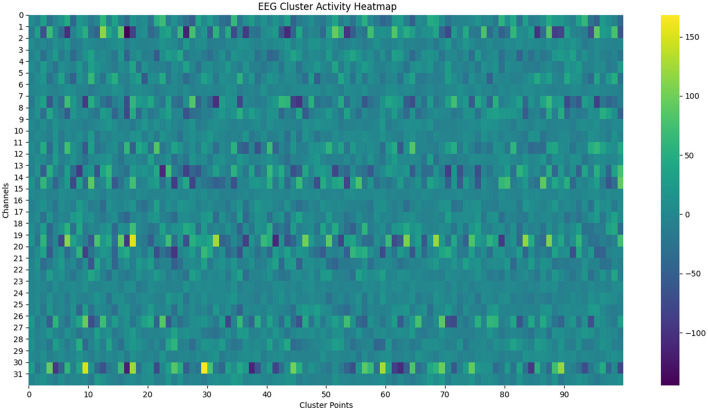
EEG cluster activity heat map, it shows the activity levels of 32 EEG channels at different clustering points (from 0 to 290).

As shown in the Figures 2, 3, we observe the audio feature map and the EEG feature map. Figure 2 displays the variation of Mel-frequency cepstral coefficients (MFCC) over time in paragraph 0. Time (s) is on the horizontal axis, MFCC coefficients are on the vertical axis, and the color bar represents the range of coefficient values, with red indicating positive values and blue indicating negative values. The intensity of the color represents the magnitude of the absolute value. The figure clearly shows the variation of spectral features of the audio signal over time, with red regions indicating periods of higher MFCC coefficients and blue regions indicating periods of lower coefficients. These features reveal the spectral characteristics of the audio signal at different time points, aiding in the intuitive observation of audio content and properties. Figure 3 illustrates the heat map of EEG cluster activity, showing the activity levels of 32 EEG channels at different clustering points (ranging from 0 to 290). Clustering points are on the horizontal axis, EEG channels on the vertical axis, and the color bar represents the range of activity values: yellow indicates high activity, blue indicates low activity, and green indicates activity close to zero. The distinct changes in activity levels of different channels at different clustering points reflect the dynamic characteristics of EEG signals. These changes reveal the activity patterns of the brain under different time or experimental conditions, aiding in further analysis of brain function and characteristics. The graphical features of EEG signals and audio are learned from two-dimensional images using CNNs and transformers. Our method is capable of learning universal latent features within complex patterns and effectively mapping these features for both relationships. From these two figures, it can be observed that there is a certain correspondence between the feature variations in the audio signal Figure 2 and the variations in EEG activity Figure 3, indicating that different features of the audio signal may elicit different responses in the EEG waves.

### 4.5 Ablation study

We conducted extensive ablation experiments based on the characteristics of the model. We built CNN, transformer, LSTM, and complete models evaluated under k=1 and 10, with ablations for four clustering approaches: without using EEG clustering $w/o\;F_{DBSCAN}^{EEG}$, without using audio clustering $w/o\;F_{DBSCAN}^{M}$, without using both clustering methods $w/o\;F_{DBSCAN}^{EEG}$ and $w/o\;F_{DBSCAN}^{M}$. Based on Table 3, our complete model demonstrates the best experimental performance. Comparing different methods, the complete model combining CNN and transformer shows performance improvements. CNN is utilized to extract important hidden features corresponding to EEG and emotional music in ${\text{F}}_{EEG}^{F}$ and ${\text{F}}_{M}^{F}$. Through ablation experiments on these two clustering methods, it is indicated that our method can effectively cluster and encode EEG and music data, thus constructing effective discrete representations. Our model achieved the best results in the experiments, with a score of 24.27% at *k* = 1 and 57.24% at *k* = 10. In the complete model combining CNN and Transformer, the CNN effectively extracts hidden features in EEG and music because CNN excels in processing temporal and spatial data. The Transformer, on the other hand, captures long-distance dependencies between data, improving the overall performance of the model. In ablation experiments, the model's performance decreased when not using the clustering method, indicating that the clustering method plays a crucial role in constructing discrete representations. These results are consistent with our expectations, as combining these two advanced deep learning techniques and clustering methods maximizes the utilization of data features, enhancing the model's prediction accuracy. Although the overall performance improved significantly, in some ablation cases, the performance decline of the model was greater than expected. For instance, the model's performance dropped significantly when the audio clustering method $w/o\;F_{DBSCAN}^{M}$ was not used. This may be because audio features play a more important role in emotional music generation, and the absence of this information makes it difficult for the model to accurately capture emotional characteristics. This suggests that future research should further optimize audio clustering methods to ensure their robustness in different scenarios. Through a series of ablation experiments varying the number of transformer layers *d*, we found that the best performance is achieved when *d* = 12, as shown in Table 4. Specifically, as the number of layers increases, the model's performance initially improves but then levels off. The table indicates that when *d* = 12, the model's performance reaches its highest value of 24.27 and 57.24%. This result suggests that, within the experimental setup, increasing the number of layers can enhance model performance to a certain extent, but performance begins to slightly decline beyond 12 layers. This may be due to the redundancy of parameters in excessively deep models, which can lead to overfitting.

**Table 3:** Conducting ablation studies of *Hits@k* for different models on the DEAP dataset.

**Model**	**Evaluation**	** $w/o\;F_{DBSCAN}^{EEG}$ **	** $w/o\;F_{DBSCAN}^{M}$ **	**$w/o\;F_{DBSCAN}^{EEG}$ and $w/o\;F_{DBSCAN}^{M}$**	**Using**
CNN	***Hits@*1(%)**	9.21	7.43	4.71	12.74
	***Hits@*10(%)**	14.23	13.79	10.23	26.23
Transformer	***Hits@*1(%)**	12.93	11.42	5.64	21.89
	***Hits@*10(%)**	24.23	25.34	12.31	51.12
LSTM	***Hits@*1(%)**	19.74	16.34	9.34	20.13
	***Hits@*10(%)**	41.13	30.35	15.12	46.18
Ours	***Hits@*1(%)**	23.34	8.23	5.62	**24.27**
	***Hits@*10(%)**	43.68	23.12	16.12	**57.24**

Bold text indicates the best values.

**Table 4:** The impact of different numbers of encoding layers and decoding layers *d* in the transformer on our model's performance.

**d**	**8**	**10**	**12**	**14**	**16**
***Hits@*1(%)**	22.48	23.74	**24.27**	24.01	23.83
***Hits@*10(%)**	53.40	56.33	**57.24**	56.97	56.22

Bold text indicates the best values.

### 4.6 Limitations

Current research on the correspondence between music and EEG signals faces significant limitations in terms of limited data, the need for complex preprocessing, and discrepancies in signal correspondence.

- Limited data on music and EEG correspondence: Currently, there is a scarcity of data regarding the correspondence between music and EEG signals, which limits the breadth and depth of research. Due to the diversity and complexity of music, as well as individual differences in EEG signals, collecting and analyzing a sufficient number of data samples is a significant challenge. This makes it difficult to draw generalizable conclusions under different experimental conditions, limiting a comprehensive understanding of the relationship between music and EEG signals.
- Need for preprocessing of music data: Different types and styles of music data require complex preprocessing, which increases the difficulty of research and application. Music data often contains a large amount of information, including melody, harmony, rhythm, and more, which needs to be accurately extracted and processed. Additionally, different styles of music (such as classical, pop, electronic music, etc.) have significant structural and characteristic differences, requiring researchers to develop diverse preprocessing methods to ensure the quality and consistency of the data.
- Discrepancies in discrete correspondence between music and EEG signals: There may be discrepancies in the discrete correspondence between EEG signals and music, leading to issues in testing emotional consistency and affecting the accuracy and reliability of the research. EEG signals are inherently highly dynamic and complex, influenced by various external factors such as the psychological state of the subject and environmental noise. Emotional responses elicited by music are also multidimensional and subjective, making it challenging to accurately match EEG signals with music stimuli in experiments. These discrepancies can lead to errors in testing emotional consistency, affecting the validity of research results.

### 4.7 Future directions

Future research should focus on the generation of multimodal emotional music, the construction of an evaluation system for individual differences, and the systematic construction of experiments and emotional datasets.

- Generation of multimodal emotional music: Explore the use of other features (such as visual, linguistic, tactile, etc.) as supplementary information to enhance the expressiveness of music data. By integrating multimodal data, the emotional expression of music can be supplemented and enriched. For example, combining visual arts (such as videos or images) with music, or combining the emotional content of lyrics with the melody and rhythm of music, to form a more complete emotional experience. This multimodal data fusion approach is expected to improve the quality and depth of emotional music generation.
- Construction of an evaluation system for individual differences: Research and understand the different interpretations of the same music by different individuals, and establish standards for the variation in generated music. Build a system that includes both subjective and objective evaluations to more comprehensively assess the effectiveness of music generation. Subjective evaluations can be obtained through user feedback, surveys, and other methods, while objective evaluations can rely on emotional recognition algorithms, EEG signal analysis, and other technical means. Such an evaluation system will help to more accurately adjust and optimize music generation models to meet the emotional needs of different users.
- Systematic construction of experiments and emotional music datasets: Conduct systematic experiments to collect and organize emotional music datasets. This includes recording subjects' EEG signals and physiological responses in controlled environments while collecting their emotional feedback on different music segments. The dataset should cover various music types and emotional states to provide rich data support for emotional music generation and analysis. By constructing high-quality emotional music datasets, the development of emotional music research can be promoted, providing a solid foundation for model training and validation.

## 5 Conclusions

This research introduces a technique that employs clustering to create a discretized representation and uses a Transformer model to reverse the mapping relationships. This method generates music imbued with subjective emotions from intricate, continuous EEG data. Our approach has demonstrated notable improvements in performance over previous methods. Extensive experiments and evaluations have confirmed the model's efficacy and robustness in producing music that expresses emotions from brain signals, highlighting its potential applications in interdisciplinary areas such as affective computing and music generation.

Next, we plan to delve deeper into the generalizability and scalability of our proposed method across various EEG datasets and music genres. We will also examine the interpretability of the music generated and its correlation with the underlying emotional states encoded in the EEG signals. By incorporating a real-time feedback mechanism, we aim to better respond to users dynamic emotional states and facilitate interactive music generation. This exploration will address the individual differences in brainwave patterns during emotional expression and enhance our understanding of the cognitive and emotional processes underlying music perception and emotional regulation.

Looking ahead, we anticipate the emergence of innovative applications for AI-driven music generation systems that can perceive emotions, enhancing human experience and wellbeing. These systems are expected to integrate seamlessly into everyday settings, including healthcare, entertainment, and assistive technologies. They aim to boost emotional intelligence and foster emotional and social wellbeing. With ongoing advancements in artificial intelligence, neuroscience, and musicology, new pathways involving creativity, expression, and human-computer interaction are expected to open up, ushering in the era of more accessible human-computer emotional interactions.

## Data Availability

The raw data supporting the conclusions of this article will be made available by the authors, without undue reservation.
